# Gas embolism during surgical hysteroscopy leading to cardiac arrest and refractory hypokalemia: A case report and review of literature

**DOI:** 10.1097/MD.0000000000035227

**Published:** 2023-09-15

**Authors:** Rong Xu, Xuefei Zhou, Longfei Wang, Yunfei Cao

**Affiliations:** a Health Science Center, Ningbo University, Ningbo, China; b Department of Anesthesiology, Beilun District People’s Hospital of Ningbo, Ningbo, China; c Department of Anesthesiology, Qilu Hospital (Qingdao), Cheeloo College of Medicine, Shandong University, Qingdao, China.

**Keywords:** Cardiac arrest, Gas embolism, Refractory hypokalemia, Resuscitation, Surgical hysteroscopy

## Abstract

**Rationale:**

One of the catastrophic complications of surgical hysteroscopy is venous gas embolism (VGE), and this event could cause morbidity and in serious cases may even lead to death. However, in cases of VGE accompanied by refractory hypokalemia is rare and can significantly increase the difficulty of treatment and resuscitation. Here, we successfully treated a patient with fatal VGE during surgical hysteroscopy, accompanied by difficult resuscitation with refractory hypokalemia.

**Patient concerns:**

We report a rare case of sudden cardiac arrest due to VGE during surgical hysteroscopy, followed by difficult resuscitation with refractory hypokalemia.

**Diagnosis:**

VGE was diagnosed by a sudden decrease in EtCO_2_, a loud mill wheel murmur in the thoracic area, and a small number of air bubbles evacuated from the internal jugular catheter. And refractory hypokalemia was diagnosed by serum potassium levels dropping frequently to as low as 2.0 mmol/L within 36 hours of resuscitation after cardiac arrest.

**Interventions:**

Our vigilant anesthesiologist noticed the early sign of VGE with a sudden drop in EtCO_2_, and as the cardiac arrest occurred, interventional maneuvers were implemented quickly including termination of the surgical procedure, adjustment of the patient’s position, cardiac resuscitation, continuous chest compression, and correction of electrolyte disturbances, particularly refractory hypokalemia during the early stage of resuscitation.

**Outcomes:**

The patient regained consciousness 4 days after the cardiac arrest and was discharged 1 month later without any neurological deficits.

**Lessons:**

As a relatively simple procedure, surgical hysteroscopy may have catastrophic complications. This case demonstrates the full course of fatal gas embolism and difficult resuscitation during hysteroscopic surgery, and emphasizes the importance of early detection, prompt intervention, and timely correction of electrolyte disturbances, such as refractory hypokalemia.

## 1. Introduction

Hysteroscopy is a minimally invasive surgical technique used for diagnostic and therapeutic interventions in the treatment of intrauterine pathology. While the procedure is generally considered safe and popular, even in primary hospitals, it may potentially cause serious complications such as venous gas embolism (VGE).^[1,2]^ Depending on the detection method used, the incidence of VGE varies from 10 to 50%, and the manifestations of this complication may range from clinical imperceptibility to the need for resuscitation.^[3]^ While the chance of VGE has decreased significantly since the introduction of liquid distension medium a decade ago. However, there is still a 0.03% risk of serious VGE that may result in death, and meanwhile, the incidence of subclinical VGE events during operative hysteroscopy is often significantly underestimated.^[4]^ As such, this catastrophic iatrogenic complication still makes what is otherwise a relatively simple procedure quite risky, and current opinions emphasize that strict vigilance and adequate monitoring are of paramount importance in detecting VGE early and avoiding a fatal outcome.^[3,4]^ However, in cases of VGE accompanied by refractory hypokalemia, even when clinical signs of VGE are detected in time and active intervention is taken, it is often very difficult to reverse the severe gas embolism and prevent catastrophic consequences. Here we report a rare case of cardiac arrest induced by VGE during surgical hysteroscopy, followed by difficult resuscitation with refractory hypokalemia.

## 2. Case presentation

The patient, a 47-year-old woman (ASA I) with no significant medical history, was admitted for treatment of intrauterine neoplasm and a right ovarian cyst, and scheduled for laparoscopic ovarian cystectomy and hysteroscopic metroplasty. On September 18 at 8:20 am, the patient was administered total intravenous anesthesia (sufentanil, propofol, remifentanil, and atracurium), with standard noninvasive monitoring. Mechanical ventilation was delivered with oxygen in air without nitrous oxide (FiO_2_ = 0.5), resulting in an arterial oxygen saturation (SaO_2_) of 100%, as measured by pulse oximetry, and an end-tidal CO_2_ (EtCO_2_) of 4.36 kPa. Then surgical procedure began at 8:45 am, and the right ovarian cystectomy was completed 55 minutes later, After another 40 minutes later, the patient was placed in reverse Trendelenburg position, and uterus was distended with glycine at a pressure of 50 mmHg. Around 5 minutes after starting hysteroscopic metroplasty, a sudden decrease in EtCO_2_ was observed, from 4.36 to 3.2 kPa in <1 minute. However, peripheral oxygen saturation was still acceptable at 96%, and no significant changes in electrocardiogram (ECG) and noninvasive blood pressure (NIBP) were detected at that time.

**Figure 1:**
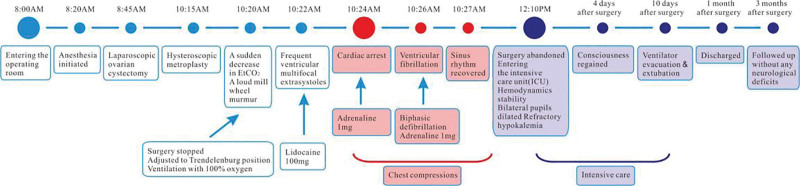
The whole process of the occurrence and treatment of venous gas embolism.

Possible causes such as hypovolaemia, ventilatory changes, and artifacts were excluded, and a VGE was suspected, A loud mill wheel murmur was heard periodically with a stethoscope, confirming the diagnosis of a large VGE. Treatment was immediately initiated, surgery was halted, and the patient was placed in Trendelenburg position to prevent further gas entrainment. Ventilation was maintained with 100% oxygen. About 30 seconds later, frequent ventricular multifocal extrasystoles were detected on ECG, and lidocaine 100 mg was injected intravenously. However,1 minute later, cardiac arrest occurred at 10:24 am, and chest compressions were immediately initiated. At the same time, adrenaline 1 mg was injected intravenously, but no normal sinus rhythm was restored. About 1.5 minutes after cardiac arrest, the patient developed ventricular fibrillation, and biphasic defibrillation with 150 J was performed. Chest compressions were continued, and another adrenaline 1 mg was administered intravenously 3 minutes later, which restored sinus rhythm with a heart rate of up to 140 beats per minute. However, the noninvasive blood pressure remained low (62/21 mmHg), and hypotension was treated with rapid intravenous saline infusion, adrenaline (40–300 mg/h) injection, and continuous chest compressions (lasting for about 30 minutes after cardiac arrest) to maintain hemodynamic stability. A 7-F central venous catheter was then inserted into the internal jugular vein to retrieve gas, and 3 attempts were made to evacuate gas from the central vein (30 mL blood per aspiration), only approximately 0.5 mL of bubbles was removed. Left iliac artery was also catheterized for invasive blood pressure (IBP) monitoring and blood gas analysis. Blood gas analysis revealed a pH of 7.30, PCO_2_ 55 mm Hg, PO_2_ 185 mm Hg, base excess –0.1 mmol/L, O_2_ saturation 100%, Hct 33 g/dL, HCO_3_^-^ 27.1mEq/L, Na^+^ 146 m mol/L, K^+^ 2.0 mmol/L, and Glu 9.8 mmol/L. 2 g (26.8 mEq) potassium chloride were supplemented within 1.5 hours via central venous. At 00: 05 pm, hemodynamics had stabilized for more than 15 minutes, with heart rate maintained at 90–100 beats per minute and IBP at around 100/60 mm Hg. Only bilateral pupils were dilated, with a diameter of approximately 4.5 mm and no light reflection was observed at that point. Surgery was abandoned, and the patient was transferred to the intensive care unit (ICU). Cardiac evaluation was normal, and a chest X-ray revealed multiple exudative changes in both lungs. Subsequent intensive care included hypothermic brain protection, respiratory support, dehydrating agent (mannitol), diuretic (furosemide), high-dose hormone (methylprednisolone), antioxidant (edaravone), neurotrophic drugs (gangliosides), antibiotics and vasoactive drugs. Around 8: 00 pm that night, the patient developed a seizure, which was considered as secondary epilepsy. Valproic acid was used for antiepileptic treatment, and a head CT scan showed that the anterior lobes of the forehead were shallower, suggesting brain swelling. The whole process of the occurrence and treatment of VGEs was shown in Figure 1.

A notable aspect of this case is the presence of refractory hypokalemia, with serum potassium levels dropping frequently to as low as 2.0 mmol/L, and a total of 13 g (174.2 mEq) of potassium chloride (excluding the 2 g used in the operating room) was administered over 36 hours after admission to the ICU. Although the planned hysteroscopy surgery was aborted due to the patient’s cardiac arrest, the intrauterine neoplasm (later confirmed as intrauterine polyps through pathological examination) was largely removed during the procedure, and subsequent ultrasound examination revealed no obvious tumor residue in the uterine cavity. Therefore, no further surgical or other interventions were required to address the uterine issue. The patient regained consciousness 4 days after the cardiac arrest and was successfully extubated after ten days of mechanical ventilation. Chest and skull CT scans showed no abnormalities. The patient made a full recovery and was discharged 1 month later. A 3-month follow-up revealed no neurological deficits. The changes of refractory hypokalemia after cardiopulmonary resuscitation were shown in Table 1.

**Table 1:** Temporal relationship between laboratory values and potassium supplement.

Date and time	09/17 10:21	09/18 9:05	09/18 10:50	09/18 13:30	09/18 22:05	09/19 04:55	09/19 11:08	09/19 17:11	09/19 23:57
Sodium (mmol/L)	141.5	139	146	141	150	151	141	147	157
Potassium (mmol/L)	3.88	3.7	2.0	2.7	2.1	2.0	2.7	2.6	3.6
Chloride (mmol/L)	107.5	98.6	114.6	102.3	119.5	125.6	110.2	105.6	121.3
Bicarbonate (mmol/L)	None	27.9	27.1	21	13	11.4	15	11	18
Glucose (mg/dL)	5.85	6.6	9.8	8.7	13.3	10	8.4	10.7	7.2
Hct(%)	35	33	29	38	40	39	35	34	32
pH	None	7.43	7.30	7,29	7.12	7.08	7.18	7.12	7.25
pCO_2_	None	43	55	44	39	50	41	33	40
Base excess (mmol/L)	None	4.2	-0.1	-5.3	-16.0	-15.3	-12.5	-17.6	-9.2
Cumulative potassium received (mEq)	0	0	26.8	53.6	93.8	134	160.8	187.6	201
Patient events	Preoperative	During laparoscopy	25minutes after arrest	85minutes after entering ICU	10hours after entering ICU	17hours after entering ICU	23hours after entering ICU	29hours after entering ICU	36hours after entering ICU

The patient’s laboratory values and blood gas analysis from preoperative ward, through her cardiac arrest, and then on a full resolution of hypokalemia in relation to the total amount of potassium supplemented.

## 3. Discussion

While EtCO_2_ is a sensitive monitoring indicator for gas embolism, it is often not sufficient to prevent severe VGE and its pathological consequences when there is a sudden and dramatic drop in EtCO_2_ levels.^[5]^ This case illustrates that even with vigilant anesthesiologists who promptly detected the abnormal decrease in EtCO_2_ and implemented interventional maneuvers, the resuscitation process was still challenging and prolonged. The most sensitive and reliable method for detecting gas embolization in the clinic is Cardiac Doppler, which can effectively predict the occurrence of gas embolism during hysteroscopy.^[6]^ However, Cardiac Doppler is not commonly used in hysteroscopic surgery due to its high cost, and most current opinions suggest that anesthesiologists should be particularly attentive when EtCO_2_ drops more than 5 mm Hg.^[4]^

There are several essential interventions for treating gas embolism. Corrective interventional maneuvers include: (1) maintaining circulation, (2) using volume expanders, and (3) preventing further gas entry.^[7]^ Once symptoms of VGE are observed, the affected patient must be immediately placed into left lateral decubitus position (right-side up) to prevent right ventricular outflow obstruction by the airlock. This is because a steep Trendelenburg position can cause negative pressure in the pelvic veins that facilitate the ingress of gas into the systemic circulation.^[8]^ Administering up to 100% oxygen can decrease bubble size by establishing a diffusion gradient that favors gas elimination, and central venous catheter placement may be necessary to effectively evacuate gas.^[5,8]^ However, attempting to retrieve gas from the right ventricle outflow through a central venous catheter is not effective in this case due to the respective delay (approximately 30 minutes after cardiac arrest). Nonetheless, about 0.5 mL of gas foam pumped out is still helpful in the diagnosis of VAE.

The importance of continuous chest compression in successfully managing these situations should not be overlooked. Continuous chest compression helps break gas bubbles in the heart chamber, increasing the right ventricular volume and restoring ventricular function to some extent.^[5]^ This resuscitation may benefit the patient in this case.

In this case, the difficulty in resuscitation may be related to concurrent refractory hypokalemia, which persisted for up to 36 hours after surgery. This unexpected finding in a patient who suffered from severe VGE and a cardiac arrest posed a challenge in managing the patient’s progressive cerebral edema during early resuscitation after cardiac arrest. Cerebral edema required treatment with hypertonic saline and mannitol, which exacerbated the already existing hypokalemia.^[9]^ While hyperkalemia is more common after cardiopulmonary resuscitation due to the exchange of potassium ions with hydrogen ions and the movement of intracellular potassium to the extracellular space in an attempt to correct the acidosis.^[10]^ VGE-induced refractory hypokalemia is clinically rare, and no relevant literature has been reported. Given the early occurrence of abnormal blood potassium in cardiopulmonary resuscitation and the poor prognosis associated with it,^[11]^ close monitoring and timely correction of refractory hypokalemia may create favorable conditions for the patient’s complete recovery. In this case, these interventions were carefully implemented until complete and prolonged stabilization of potassium was achieved. They proved to be effective and fortunately, the patient was rescued.

It is believed that hyperbaric oxygen therapy can expedite the absorption of nitrogen in the air embolism and reduce the volume of the embolic air (Tur-Kaspa, 1990). However, the majority opinion is that hyperbaric oxygen therapy is not a necessary treatment but rather an adjunctive treatment for critical or evident cases with neurological deficits.^[12]^ This patient did not receive hyperbaric oxygen therapy but eventually made a full recovery, which supports the latter view.

## 4. Conclusion

In summary, this case report presents the full course of a fatal VGE incident and difficult resuscitation during hysteroscopy, and emphasizes the importance of early detection, prompt intervention, and timely correction of electrolyte disturbances, such as refractory hypokalemia.

## Author contributions

**Conceptualization:** Yun-fei Cao.

**Funding acquisition:** Yun-fei Cao.

**Project administration:** Rong Xu, Yun-fei Cao.

**Writing—original draft:** Rong Xu, Xue-Fei Zhou, Long-Fei Wang.

**Writing—review & editing:** Yun-fei Cao.
